# Decreased Hepcidin Levels Are Associated with Low Steady-state Hemoglobin in Children With Sickle Cell Disease in Tanzania

**DOI:** 10.1016/j.ebiom.2018.07.024

**Published:** 2018-07-25

**Authors:** Nathaniel Lee, Julie Makani, Furahini Tluway, Abel Makubi, Andrew E. Armitage, Sant-Rayn Pasricha, Hal Drakesmith, Andrew M. Prentice, Sharon E. Cox

**Affiliations:** a School of Tropical Medicine & Global Health, Nagasaki University, Nagasaki, Japan; b Sickle Cell Programme, Muhimbili University of Health & Allied Sciences, Dar-es-Salaam, Tanzania; c Department of Haematology & Blood Transfusion, Muhimbili University of Health and Allied Sciences, Dar-es-Salaam, Tanzania; d MRC Human Immunology Unit, Weatherall Institute of Molecular Medicine, University of Oxford, UK; e London School of Hygiene and Tropical Medicine, London, UK

**Keywords:** Sickle cell disease, Nutrition, Iron metabolism, Hepcidin, Sickle cell anemia, Sub-Saharan Africa

## Abstract

**Background:**

The contribution of hepcidin as a regulator of iron metabolism & erythropoiesis on the severity of anemia in sickle cell disease (SCD) remains poorly characterized, especially in Sub-Saharan African populations. The aims of the study were to determine if hepcidin is associated with severity of steady-state anemia in SCD and to investigate factors associated with hepcidin and anemia in SCD.

**Methods:**

Archived samples from 199 Tanzanian children, 56% boys aged 3–18 with laboratory-confirmed SCD were analysed based on recorded averaged steady-state hemoglobin (ASSH) quartiles (lowest vs. highest). Univariable and multivariable logistic regression was used to assess associations with ASSH quartiles.

**Findings:**

In univariable analysis, hepcidin <5·5 ng/mL was associated with increased odds of being in the lowest ASSH quartile (OR 2·20; 95%CI 1·2–3·93) but which was limited to girls (OR 4·85, 95%CI 1·79–13·09, *p* = .046 for interaction). In multivariable analyses including either reticulocyte percentage or erythropoietin, lower hepcidin remained significantly associated with lowest ASSH quartile, although the hepcidin-sex interaction no longer reached statistical significance. No associations with ASSH quartile were observed for markers of inflammation, hemolysis or potential iron markers except for microcytosis, associated with higher ASSH, but which was confounded by reticulocyte percentage and alpha-thalassaemia status.

**Interpretation:**

Hepcidin is lower in more severely anaemic children with SCD independent of inflammation or markers of erythropoiesis.

**Funding:**

Funding sources include The Wellcome Trust (080025, 095009, 094780 & 070114), MRC-UK (MC-A760-5QX00), NIHR Oxford Biomedical Research Centre, and the Bill and Melinda Gates Foundation (“Hepcidin and Iron in Global Health”, OPP1055865).

## Highlights

• Low hepcidin levels are associated with severe anemia independent of markers of erythropoiesis and inflammation.
• Low serum ferritin is present in some children but is not associated with severe anemia.
• Although iron markers remain difficult to interpret in sickle cell disease, hepcidin levels were mostly below levels that would be expected to inhibit iron absorption and recycling.

**Research in Context**

Sickle cell disease (SCD) is one of the most common inherited disorders globally, affects hemoglobin production, and is a major cause of child mortality and poor health, most prominently in Sub-Saharan Africa. Anemia is a defining feature of SCD, but the relative causes of the degree of severity are incompletely understood. Hepcidin downregulates iron absorption and supply to tissues in inflammation and infection and levels decrease when iron is limited or red cell production is increased or under low oxygen conditions. In this study, we show that low hepcidin was associated with severe anemia in children with SCD, independent of markers of inflammation or erythropoietic drive and that this effect appeared to be limited to girls.

Alt-text: Unlabelled Box

## 1 Introduction

Sickle cell disease (SCD) is one of the most common inherited diseases with the majority of the burden occurring in Sub-Saharan Africa and India, settings where iron deficiency is also common. [1] SCD has a range of clinical manifestations, including anemia from increased hemolysis. [2] SCD is also associated with an increased risk of mortality, contributing significantly to under-five mortality in Sub-Saharan Africa. [3]

Low iron status has not been considered a significant contributor to the degree of severity of anemia in SCD as it is assumed that iron absorption would increase to meet the increased erythropoietic need. Furthermore, as children with SCD may receive repeated blood transfusions, iron overload is more of a concern. [4] An increased understanding of the regulation of iron metabolism, and in particular the role of hepcidin a 25-amino-acid peptide hormone synthesized by hepatocytes as a key negative-feedback regulator of iron status, suggests that sickle-related processes of increased erythropoiesis and inflammation may have conflicting effects on hepcidin and hence iron status. [5] Hepcidin blocks iron absorption across the gut and iron efflux from the reticulo-endothelial system. Hepcidin expression is increased by inflammation and results in a hypoferremic state, which is an important anti-infective response. [6] Hepcidin expression is decreased by erythropoiesis and hypoxia, thereby increasing iron absorption and release of body iron to meet increased erythron demand. [6]

The contribution of iron deficiency to anemia in patients with SCD living in areas where nutritional iron deficiency anemia is common is unknown. The measurement of iron status is complicated by chronic inflammation and increased erythropoiesis that confound biomarkers of iron status. [7] The relative, quantitative effects of these processes compared to iron status on iron markers are not known. Limited data regarding sensitivity and specificity of iron markers in SCD exist. Low serum ferritin is specific for iron deficiency but in SCD has low sensitivity due to effects of inflammation. [8] Transferrin saturation is decreased in iron deficiency but can also be decreased by the hypoferremic acute phase response to infection and inflammation. The gold standard method of iron staining in bone marrow biopsies is rarely conducted; but in one small study in 60 Indian SCD adult patients, 28% were reported to have absent stainable iron in bone marrow aspirates. Importantly, although the specificity of ferritin <30 ng/mL for iron deficiency by bone marrow was 99%, the sensitivity was only 32%, indicating iron deficiency may be present at higher ferritin concentrations in this condition. [8]

The severity of anemia in SCD may also be affected by the *co*-inheritance of other SCD modifying polymorphisms, including the α-thalassaemia 3·7 deletion and glucose-6-phosphate dehydrogenase (G6PD) deficiency [9]. Co-inheritance of α-thalassaemia in SCD modifies red cell indices and results in decreased hemolytic markers. [10] In some reports, it has also been associated with effects on total hemoglobin and steady-state hemoglobin level. [11] G6PD deficiency results in decreased capacity to reduce oxidized glutathione via NADPH, and thus the reduced ability of red cells to counteract oxidant stress. [10] G6PD deficiency (A-genotype) in sickle cell anemia is associated with lower hemoglobin but not increased hemolysis. [12]

Iron deficiency in non-SCD populations is associated with reduced cognitive development and function and in SCD may increase the severity of anemia, with consequent reduction in quality of life and survival. [13,14] Paradoxically, there are reports that patients with SCD in iron-deficient states may have better SCD-related clinical outcomes. [15,16] Levels of hemolysis and steady-state hemoglobin level have been used to differentiate severity of disease in SCD. [17]

Here, we sought to determine if hepcidin is associated with lower or higher average steady-state hemoglobin (ASSH) level in children with SCD; and to evaluate how such an effect might be mediated.

## 2 Methods

### 2.1 Study Design and Population

The study was conducted in children enrolled in the Muhimbili Sickle Cohort (MSC) in Dar-es-Salaam, Tanzania who regularly attended the outpatient clinic between 2006 and 2009. [14] Samples were selected for analysis from children in the lowest or highest average (over 12 months) steady-state hemoglobin (ASSH) quartile within their age group (HbQ1 or HbQ4). Selecting comparison populations based on usual hemoglobin status, rather than hemoglobin on a single day, reduced potential variability. Prospectively collected and archived steady-state plasma and serum samples were selected as per the criteria outlined below from children aged 3–18 years on the day of sample collection. At that time, routine penicillin prophylaxis and pneumococcal vaccination had not been implemented and no children were on regular blood transfusions. Venous blood for blood counts and peripheral oxygen saturation (SpO_2_) using pulse oximetry (Nelcor Haywood, CA or Masimo Radical, Irvine CA, USA) were routinely collected at these clinic visits. The definition of steady-state for selection of blood samples to be assessed for the laboratory investigations and for the calculation of ASSH was defined as a routine scheduled outpatient clinic visit in the absence of recorded pain, fever (temperature > 37·4 °C), or current/recent malaria infection (malaria rapid test or slide positive). Samples were further excluded if hospitalization was known to have occurred within a month of sample collection, or were probable HbSβ^+^. Otherwise, all individuals were HbSS diagnosed by quantification of hemoglobin fractions performed by high performance liquid chromatography (HPLC) using the β-thalassaemia Short Program on the Variant I analyser (BioRad, Hercules, CA, USA).

#### 2.1.1 Laboratory Procedures

Blood counts (Pentra 60, Horiba ABX, Kyoto, Japan) were routinely collected on EDTA samples. Fetal hemoglobin was quantified using HPLC. At annual visits, serum samples were collected for routine clinical chemistry analyses (Roche Cobas Mira, New York or Abbott Architect, New York, USA) as part of MSC follow-up and included liver function tests; aspartate transaminase (AST), alkaline phosphatase (ALP) and direct and indirect bilirubin, plus lactate dehydrogenase (LDH) as a marker of hemolysis. Remaining plasma and serum samples were archived at −80 °C. Serum ferritin, serum iron, transferrin, C-reactive protein (CRP) and α-1 acid glycoprotein (AGP) were measured in archived samples in batches on an automated analyser (Roche Cobas Mira, New York). Plasma erythropoietin (EPO) and soluble transferrin receptor (sTfR) were measured using ELISA (R&D systems) as per manufacturer's instructions. Plasma hepcidin was measured with a competitive ELISA (Hepcidin-25 [human] EIA Kit, Bachem) by a trained laboratory scientist using an adapted method as published elsewhere. [18] Hepcidin:transferrin saturation and hepcidin: ferritin ratios were calculated as surrogate measures of hepcidin expression on circulating and stored iron respectively, as previously described [19,20]. All samples were analysed in the Muhimbili Sickle Cohort research laboratory in Muhimbili National Hospital, Tanzania. Standards and samples were analysed in duplicate or triplicate (hepcidin). Samples with readings outside the standard curvilinear region were repeated at appropriate dilutions. Sample results with a coefficient of variation over 10% (≥12% for hepcidin) were repeated.

Co-inheritance of potential SCD modifying genotypes including α–thalassaemia genotype for the 3·7 deletion and The 202- and 376-single nucleotide polymorphisms (SNPs) (rs1050828 [G-202A] & rs1050829 [A-376G]), the combined inheritance resulting in the A- phenotype of glucose 6-phosphate dehydrogenase (G6PD) deficiency were assessed as previously reported. [9]

#### 2.1.2 Statistical Analysis

Data were analysed using Stata IC (StataCorp LP v14·1). Adjustment for within individual clustering for repeated samples was accounted for using robust standard errors. Statistical significance was set as *p* ≤ 0·05. Missing data of ≥5% per variable with a pattern of missingness that was random or completely random was imputed using chained equations and *n* = 20 imputation sets. [21]

Univariable analysis accounting for repeated observations in some individuals was performed using logistic regression. Results were reported as odds ratios with associated 95% confidence intervals. Continuous variables were checked to ensure that they fulfilled linearity assumptions on regression. If this was not the case, then a strategy of transformation using fractional polynomials was employed in order to avoid categorizing variables. [22] Results were scaled as necessary to present interpretable data. Dataset was also tested for possible effect modifications.

Multivariable analysis was performed, with a variable selection strategy stratified to include at least one variable from each of the major biological processes known to influence SCD processes. This stratification included variables describing a general inflammatory state, iron metabolism, and reticulocyte percentage or serum erythropoietin as a marker of erythropoietic drive. Logistic regression was performed as the primary multivariable modelling tool, adjusted for age and sex.

#### 2.1.3 Ethics Statement

The study received ethical approval from Muhimbili University of Health and Allied Sciences reference MU/RP/AEC/VOL XI/33) and the London School of Hygiene & Tropical Medicine (reference 5158). Informed consent was obtained from patients or guardians at screening and at enrolment into the cohort.

#### 2.1.4 Role of the Funding Sources

The stated funding sources did not have any role in the in study design; in the collection, analysis, and interpretation of data; in the writing of the report; or in the decision to submit the paper for publication.

## 3 Results

**Fig. 1:**
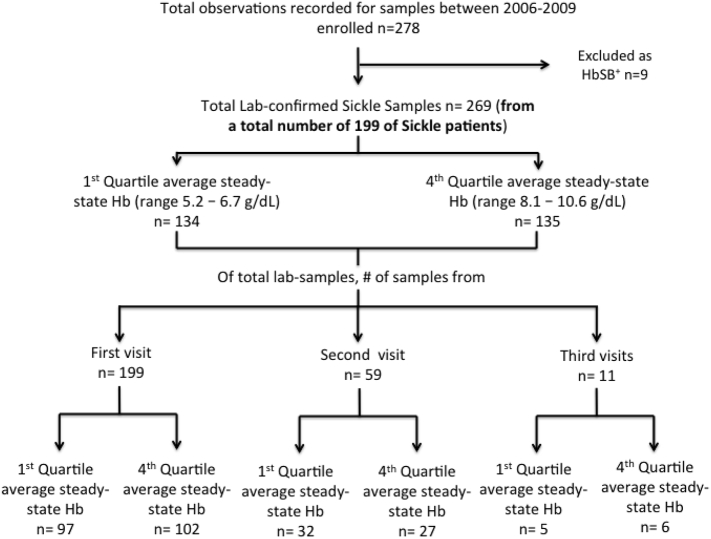
Flow chart of study population and sample analysis.

A total of 269 samples were analysed from 199 children (Fig. 1). Fifty-nine children had a second sample, and 11 children had a third sample included in the analysis. The range of hemoglobin for ASSH levels in the two quartile groups was 5·2–6·7 g/dL in the HbQ1 group and 8·1–10·6 g/dL in the HbQ4 group (Fig. 1). Hemoglobin assessed at the same timepoint as the biochemical measurements was outside the ASSH range for 8% of those in HbQ4 and 9% of those in HbQ1 group.

Univariable analysis of the two ASSH quartile groups by age, sex, markers of potential iron status, red cell indices, markers of inflammation and hemolysis, erythropoietin, hepcidin, SpO_2_, α-thalassemia genotype, G6PD status, and fetal hemoglobin level are shown in Table 1. Boys were significantly more likely to be in the more severely anaemic HbQ1 group (OR 2·40; 95%CI 1·28–4·50), but age was not associated. Inflammatory markers did not differ between ASSH quartile groups. None of the measured potential iron markers were associated with particular ASSH quartiles. The proportions of children classified as iron deficient by serum ferritin <30 g/L, were 11% and 7% in the most severely anaemic HbQ1 and least severely anaemic HbQ4 groups respectively (*p* = 0·301), compared to 16% and 15% with transferrin saturation < 16% in the HbQ1 and HbQ4 groups respectively (p = 0·574). Microcytosis was more common in the less anaemic HbQ4, 60% compared to 39% in HbQ1 (p = 0·002). The HbQ1 group was at higher odds of increased reticulocyte percentages compared to the HbQ4 group (OR 1·05, p = 0·036). The proportion of children with hepcidin levels below 5·5 ng/mL, previously shown to indicate iron deficiency and increased iron utilisation in Gambian children, was significantly higher at 73% in the most anaemic HbQ1 group compared to 55% in HbQ4 group (*P* = 0·007). [18] Both the hepcidin/ferritin and hepcidin/transferrin saturation ratios were lower in the HbQ1 group (OR 0.72, *p* = 0.003 and OR 0.71, p = 0.003 respectively).

**Table 1:** Factors associated with steady-state, averaged hemoglobin first and last quartile (HbQ1 & HbQ4). Univariable analysis using logistic regression for association with HbQ1, adjusting for multiple sample observations.

	Total sample observations	Low steady state Hgb (HbQ1)	High steady state Hgb (HbQ4)	OR [95% CI] *P*-value
Sex (total group *n* = 269)				
Male	150	89 (66·4%)	61 (45·2%)	2·40 [1·28–4·50] 0·006
Age category (on day of sample)	269			
1–3 years		3 (2·4%)	–	0·94 [0·60–1·47] 0·792
4–6 years		15 (11·2%)	22 (16·3%)	
7–12 years		93 (69·4%)	86 (63·7%)	
13–17 years		23 (17·2%)	27 (20·0%)	
*Inflammatory markers*				
WBC (x 10^9^/L)	264	14·4 (11·85–17·15)	12·5 (9·82–16·30)	4·80 (2·0.00–11·53) <0.001
AGP (mg/dL)	266	125·93 [97·41–158·08]	117·86 [88·85–144·72]	1·30 [0·62–2·71] 0·482^¶^
AGP elevated [>120 mg/dL]		68 (51·5%)	64 (47·8%)	1·16 [0·70–1·94] 0·566
CRP [mg/L]	266	4·19 [2·29–8·40]	3·98 [1·85–6·98]	1·12 [0·89–1·43] 0·324^¶^
CRP elevated [≥ 3 mg/L]		82 (62·1%)	84 (62·7%)	0·98 [0·57–1·68] 0·930
*Potential iron markers*				
Ferritin (μg/L)	266	116·65 [57·75–229·35]	163·15 [87·70–254·70]	0·79 [0·59–1·05] 0·102^¶^
Transferrin (g/L)	266	3·03 [2·52–3·65]	2·88 [2·53–3·54]	1·13 [0·84–1·51] 0·435
Transferrin saturation [%]	263	26·98 [18·61–40·31]	26·42 [18·98–36·14]	1·01 [0·99–1·03] 0·315
sTfR (mg/L)	259	15·01 [10·88–62·64]	13·24 [9·01–51·38]	1·00 [0·997–1·013] 0·257
sTfR-F index	256	3·42 [2·17–11·60]	2·98 [1·73–10·06]	1·02 [0·98–1·06] 0·283
Hypoferritinaemia (ferritin <30 μg/L)		15 (11%)	10 (7%)	1·59 [0·66–3·83] 0·301
sTfR-F index>5·6 [32]		51 (40·8%)	58 (44·3%)	0·87 [0·53–1·42] 0·574
Transferrin saturation < 16%		21 (16%)	20 (15%)	1·07 [0·53–2·17] 0·853
*Red cell indices*				
MCV (fL/cell)	264	84·00 [78·50–87·45]	79·00 [71·50–85·00]	1·03 [0·97–1·08] 0·323
MCHC (g/dL)	260	32·30 [31·40–33·20]	32·30 [31·50–33·20]	0·970 [0·856–1·10] 0·637
Microcytic MCV^a^		50 (38%)	79 (60%)	0·41 [0·23–0·72] 0·002
MCHC <32 g/dL		48 (37%)	51 (39%)	0·99 [0·73–1·32] 0·962
*Erythropoietic drive*				
EPO [μIU/mL]	241	110·42 [76·95–207·41]	54·96 [36·88–81·47]	1·009 [1·001–1·016] 0·016
Reticulocyte [%]	151	12.9 [9·3–16·2]	10.2 [6·2–15·8]	1·05 [1·00–1·10] 0·036
*Hemolytic markers*				
LDH [IU/L]	190	688·50 [534·50–997·50]	520·50 [377·00–908·00]	0·43 [−0·28–1·14] 0·234
Indirect bilirubin [μmol/L]	248	49·62 [24·00–83·10]	30·16 [15·83–56·60]	1·005 [0·999–1·012] 0·123
Hemoglobin oxygen saturation				
SpO_2_ (%)	217	96 [94·5–98·0]	98 [97·0–100·0]	0·72 [0·63–0·83] <0·001
Hepcidin				
Hepcidin ng/mL	255	2·00 [0·64–6·16]	4·36 [1·50–9·72]	0·74 [0·60–0·90] 0·003
Hepcidin <5·5 ng/mL		95 (73·1%)	69 (55·2%)	2·20 [1·23–3·93] 0·007
Hepcidin/Ferritin ratio	253	0.02 (0.01–0.03)	0.03 (0.01–0.06)	0.71 (0.57–0.89) 0.003
Hepcidin/Tranferrin saturation ratio	250	0.07 (0.03–0.17)	0.15 (0.07–0.46)	0.72 (0.58–0.89) 0.003
α-thalassaemia 3·7 Deletion	238			
Wild Type (αα/ αα)	93	59 (49%)	34 (29%)	
1 Deletion (−α/ αα)	96	48 (40%)	48 (41%)	0·58 [0·27–1·23] 0·155
2 Deletions (−α/− α)	49	13 (11%)	36 (31%)	0·21 [0·07–0·58] 0·003
G6PD deficiency	216			
No deficiency	171	85 (77%)	86 (81%)	
Female heterozygous	14	8 (7%)	6 (6%)	1.35 [0·39–4·62] 0·634
Male and female homozygous	31	17 (15%)	14 (13%)	1.22 [0·43–3·53] 0·702
Fetal hemoglobin (%)	228	4 [1·7–5·2]	6·7 [4·1–9·7]	0·74 [0·66–0·84] <0·001

Continuous variables are presented as medians with interquartile range in brackets. Binary or categorical variables are presented as the numerator and percentages in brackets. Variables chosen to transform using fractional polynomials fulfilled criteria of non-linearity. Normal ranges are as per international standards except for the following which are either for factors not universally harmonized or are specific to the Tanzanian child population: WBC 10^9^/L (1–5 years 3·7–13·2, 5–13 years 3·7–9·1, 13–18 years (3·2–10·3); erythropoietin 3·1–16·5 IU/L. [33, 34]  indicates variable fulfilling criteria for imputation, descriptive statistics reported for original dataset ^¶^ indicates first-degree fractional polynomial power of 0. ^a^ Cut-offs for MCV (fL) were MCV < 75 if age 1–3 years, MCV < 79 if age 3–5 years, MCV < 80 if age 6–11 years, MCV < 82 if age 11-14 years, MCV 15–74 if age 15–74 years [7].

Increased serum EPO was associated with increased odds for HbQ1 (OR 1·009; 95%CI 1·00–1·02). None of the measures of hemolysis were associated with HbQ group. A higher SpO_2_ was associated with decreased odds of being in the HbQ1 group (OR 0·72; 95%CI 0·63–0·83). Hepcidin<5·5 ng/mL was associated with increased odds of being in the HbQ1 group (OR 2·20; 95%CI 1·23–3·93). Co-inheritance of an increasing number of α-thalassaemia mutations showed a trend for decreased odds of being in the Hb1Q group with two deletions having a significant protective effect (−α/−α deletion - OR 0·21; 95%CI 0·07–0·58), whilst increased fetal hemoglobin levels were similarly associated with decreased odds (OR 0·74; 95%CI 0·66–0·84). The number of α-thalassaemia deletions was also associated with microcytosis (43% one deletion, 36% two deletions).

The results of multivariable analyses of quartile groups are shown in Table 2. Adjusted for age, sex, ferritin, inflammation and reticulocyte percentage or erythropoietin levels, low hepcidin levels were independently associated with increased odds of being in the more severely anaemic HbQ1 group. In addition, we tested for a potential interaction between low hepcidin and sex and observed a significant interaction in which the odds of low hepcidin being associated with severe anemia was limited to females (OR 4·85, 95%CI 1·79–13·09 in girls vs 1·44, 95%CI 0·71–2·92 in boys) with a Mantel Haenzel test for heterogeneity of *p* = 0·046. Although this effect remained present in the multivariable model, the interaction term was not statistically significant.

**Table 2:** Multivariable model for associations with low steady-state averaged hemoglobin quartile [HbQ1], adjusted for age and sex a-priori. Sample size of observations after imputations is *n* = 248.

Variable	Adjusted OR [95% CI] *p*-value	Adjusted OR° [95% CI] p-value	Adjusted OR (inclusive of hepcidin-sex interaction⁎) [95% CI] p-value	Adjusted OR° (inclusive of hepcidin-sex interaction⁎⁎) [95% CI] p-value
Decreased Hepcidin	2·38 [1·24–4·56] 0·009	2·27 [1·14–4·54] 0·020		
• <5.5 ng/mL in females	–	–	4·42 [1·69–11·60] 0·003	4·83 [1·40–16·60] 0·013
• <5.5 ng/mL in males	–	–	1·54 [0·62–3·87] 0·355	1·47 [0·60–3·62] 0·405
Ferritin (μg/L)a	1·001 [0·999–1·002] 0·493	1·000 [0·998–1·003] 0·776	1·000 [0·999–1·002] 0·612	1·000 [0·998–1·003] 0·886
Presence of inflammation^a^	1·11 [0·60–2·03] 0·747	0·96 [0·52–1·77] 0·893	1·06 [0·57–1·96] 0·851	0·92 [0·49–1·73] 0·805
Reticulocyte (%)^b^	1·06 [1·01–1·11] 0·028	–	1·06 [1·00–1·11] 0·033	–
Erythropoietin (μIU/mL)	–	1·01 [1·00–1·01] 0·009	–	1·008 [1·002–1·015] 0·007
Age	0.96 (0·86–1·06) 0·405	1.00 (0·90–1·12) 0·984	0·96 [0·86–1.07] 0.436	1·00 [0·90–1.12] 0.942
Male Sex	2.53 (0·06–0·93) 0·039	2.99 (1·51–5·95) 0·002	–	–
Male sex in hepcidin >5.5 (baseline group)	–	–	5·19 (1·68–16·03) 0.004	6·96 (1·77–27·35) 0.005

° Independent factor inserted into model as surrogate of erythropoiesis is erythropoietin levels.

a Presence of inflammation defined as serum CRP > 5 mg/L or serum AGP > 120 mg/L.

b Imputed continuous range of reticulocytel.

⁎ p-Value for interaction term in multivariable model is p = 0.115.

⁎⁎ p-Value for interaction term in multivariable model is p = 0.120.

**Fig. 2:**
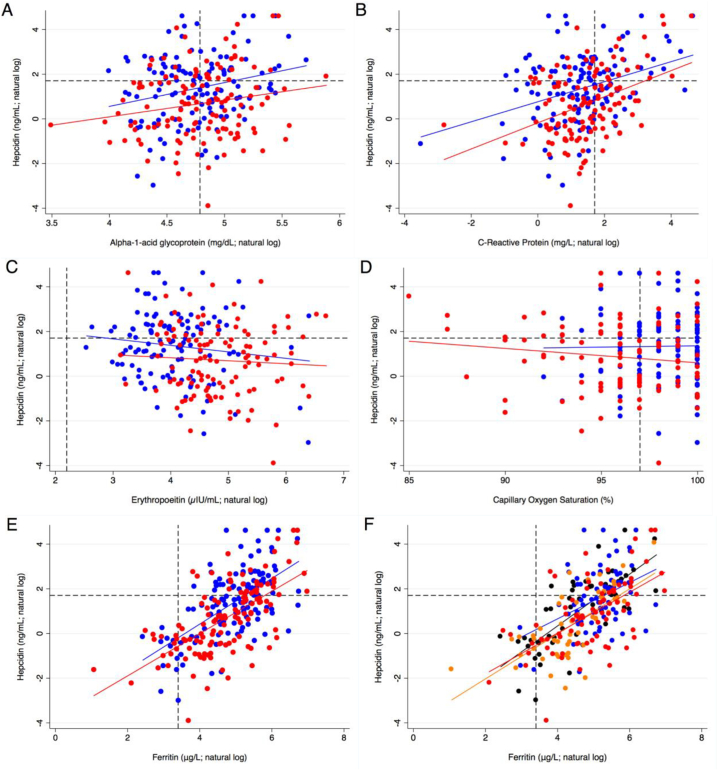
A–E - Association of serum hepcidin by ASSH quartile group with inflammatory markers: (2A and B) serum AGP and CRP; (2C) erythropoietin; (2D) Oxygen Saturation; and (2E and F) ferritin with and without inflammation. Red = low steady-state hemoglobin quartile (HbQ1); Blue = high steady-state hemoglobin quartile (HbQ4). Hepcidin cut-off demonstrated as 5.5 ng/mL. Dotted lines on the Y-axis refer to a hepcidin cut-off point at 5.5 ng/mL. Dotted lines on the X-axis are specified in the legend below. *(2A) AGP cut-off demonstrated as 120 mg/dL HbQ1 correlation coefficient 0*·*748 [95%CI 0*·*051–1*·*444], R 0*·*185, p* *=* *0*·*036; HbQ4 correlation coefficient 1*·*064 [95%CI 0*·*313–1*·*814], R 0*·*246, p* *=* *0*·*006)* *(2B) CRP cut-off demonstrated as 5 mg/L.HbQ1 correlation coefficient 0*·*585 [95%CI 0*·*345–0*·*825], R 0*·*393, p* *<* *0*·*001; HbQ4 correlation coefficient 0*·*449 [95%CI 0*·*239–0*·*658], R 0*·*359, p* *<* *0*·*001)* *(2C) Erythropoetin cut-off demonstrated at 9* μIU*/mL. HbQ1 correlation coefficient* *−* *0*·*133 [95%CI -0*·*527–0*·*261], R -0*·*063, p* *=* *0*·*505); HbQ4 correlation coefficient* *−* *0*·*291 [95%CI -0*·*682–0*·*100], R -0*·*136, p* *=* *0*·*143* *(2D) Oxygen saturation cut-off demonstrated as 97%. HbQ1* correlation coefficient − 0·064 [95%CI -0·157–0·028], r^2^ 0·018, p = 0·170); HbQ4 correlation coefficient 0·012 [95%CI -0·155–0·178], R -0·135, p = 0·889 *(2E) Ferritin cut-off demonstrated as 30 g/L. HbQ1 correlation coefficient 0*·*947 [95%CI 0*·*748–1*·*147], R 0*·*641, p* *<* *0*·*001); HbQ4 correlation coefficient 1*·*024 [95%CI 0*·*784–1*·*263], R 0*·*608, p* *<* *0*·*001* *(2F) Red = HbQ1 with systemic inflammation; Orange = HbQ1 without systemic inflammation; Blue = HbQ4 with systemic inflammation; Black = HbQ4 without systemic inflammation. Systemic inflammation defined as AGP > 120 mg/dL and/or CRP > 5* *mg/L. Without systemic inflammation - HbQ1 correlation coefficient 1*·*007 [95%CI 0*·*711–1*·*303], R 0*·*695, p* *<* *0*·*001); HbQ4 correlation coefficient 1*·*160 [95%CI 0*·*868–1*·*453], R 0*·*741, <0*·*001 With systemic inflammation - HbQ1 correlation coefficient 0*·*914 [95%CI 0*·*629–1*·*200], R 0*·*593, p* *<* *0*·*001); HbQ4 correlation coefficient 0*·*812 [95%CI 0*·*381–1*·*243], R 0*·*415, p* *<* *0*·*001*.

We investigated how hepcidin is associated with markers of known pathways affecting its expression, and whether these differed by quartile group by assessing associations between hepcidin with inflammatory markers (Fig. 2 A & B), erythropoeitin (Fig. 2C), and SpO2 (Fig. 2D). and between hepcidin and ferritin levels, in the absence and presence of inflammation (Fig. 2E & F).

There were no associations between age and sex on hepcidin concentrations. Hepcidin was positively associated with serum ferritin (r^2^ = 0·4, *P* < 0·001), which did not differ by quartile group, with similar slopes of the two regression lines. When the association between hepcidin and ferritin for each quartile group was examined in the presence or absence of inflammation, there were no apparent differences (Fig. 2 E&F). Hepcidin levels also significantly increased with increasing inflammation (CRP; r^2^ = 0·12, *p* < 0·001), and did not differ by quartile group. Hepcidin was not associated with EPO levels (r^2^ = −0·01, *p* = 0·15), with no evidence of difference between the quartile groups. Finally, there was no evidence of an association between hepcidin and SpO_2_ (r^2^ = 0·0002, p = 0·826) (Fig. 2D).

## 4 Discussion

It is important to determine the aetiology of severe anemia in SCD patients at steady-state to improve management and develop targeted interventions. We observed that low hepcidin levels were associated with more severe anemia, occurring in the absence of differences in possible iron markers between the groups. Thus, despite the limitations of iron markers in SCD, we can tentatively conclude that high hepcidin levels, and an inability to absorb and incorporate iron, are not likely to be a proximal cause of more severe anemia in this population. As expected, increased reticulocyte percentage was associated with more severe anemia but inflammation was not associated.

Hepcidin in African children without SCD has been suggested as a potential marker to indicate both low iron status, and the ability to receive and utilize iron supplements in anaemic African children. [18,23] Using the same cut off as in this study, more children in HbQ1 group had low hepcidin. Considering the proportion of children with inflammation, the proportion of children with hepcidin below this cut off was high in both groups (73% vs 55%). We do not know how hepcidin may relate to iron status in our SCD population. High levels of erythropoiesis or hypoxia may be expected to override the effect of inflammation to keep hepcidin low, but this is not supported by our observation of limited associations between hepcidin and EPO, CRP or SpO_2_. EPO might not be the best marker of erythropoietic drive as it does not take into account EPO sensitivity and therefore reticulocyte percentage may be a better indicator of erythropoietic response. However, the association between low hepcidin and anemia status was largely unchanged when adjusting for either of these markers. It remains unclear why the association between low hepcidin and more severe anemia was limited to girls. The majority of the study population were pre-pubertal, as puberty and menarche are significantly delayed in SCD, thus making it less likely that there was more true iron deficiency in girls due to menstruation. It would be instructive to measure the EPO-stimulated, erythroblast-derived hormone erythroferrone, which supresses hepcidin. Erythroferrone is increased in humans after EPO administration, and in thalassaemic patients, but levels in SCD are as yet unknown; we would hypothesise it may be elevated in the most anaemic patients if they are undergoing increased erythropoiesis. [24] There was a strong correlation between hepcidin and ferritin (r^2^ = 0·4, *p* < 0·001) and only a weak association with inflammatory markers. If hepcidin levels are low, and probably in the order of the current cut off, children should be able to absorb and utilize available iron in the diet if it were required.

The prevalence of low serum ferritin (<30 μg/L) in our total study population was 9·4%. It is possible that more children have low body iron stores than this figure suggests, as low serum ferritin is highly specific but insensitive for iron deficiency in SCD when assessed by bone marrow aspirate staining. [8] When limited to children without low-grade or severe inflammation this increased to 17·5%, similar, to the prevalence associated by transferrin saturation. In Yemeni children with SCD the prevalence of low serum ferritin was 25% when using the criteria of serum ferritin <30 μg/L if CRP < 10 mg/L, or < 70 μg/L if CRP > 10 mg/L. [25] If we used these same criteria, the prevalence of low serum ferritin in our population would be 10·5%, suggesting that these cut-offs do not adequately deal with the effect of inflammation on ferritin. The observed increased MCV and decreased proportion of microcytosis in the HbQ1 group may result from an increased rate of erythropoiesis in this group, as indicated by greatly increased EPO, and from less frequent co-inheritance of α-thalassaemia deletions which are associated with microcytosis. From the observations of the potential iron markers we can conclude that either: (i) iron deficiency although present in some children is not contributing to degree of anemia, or; (ii) these iron markers are not adequately capturing iron status in our SCD population. We propose that the 2nd option is the more likely. Dietary assessment of iron intakes might be helpful to assess if low-bioavailable iron diets are contributing to anemia severity in this population.

Few previous studies have attempted to characterize iron marker or other parameters and hepcidin in patients with sickle cell disease. Similar to our results, in a SCD cohort of adults in Holland, it was reported 5/16 (HbSS/SC & HbSβ^0^) had serum hepcidin levels below the normal range (measured by mass spectrometry, <1 mmol/L), and these tended to be in patients with lower ferritins and more severe anemia. [20] In American adults, serum hepcidin in heavily transfused SCD patients had a similar degree of correlation with serum ferritin but a larger negative correlation with EPO (r^2^ = −0·34, p-0·03), which in our dataset, did not reach statistical significance – possibly due to the extremes of hemoglobin concentration resulting from our study design. [26] Our approach of selecting samples from children at the extreme ranges of averaged steady-state hemoglobin has limitations compared to analysing samples from across the range of hemoglobin, but this design was selected as the most parsimonious in the context of the very little available information in SCD at the time.

Low averaged steady state hemoglobin levels were associated with high EPO, increased reticulocyte %, lower SpO_2_, the absence of co-inheritance of α-thalassaemia deletions, and lower levels of fetal hemoglobin. No association was seen for G6PD mutations. In Jamaican SCD patients Serjeant et al. reported α-thalassemia status, sex and fetal hemoglobin to be associated with hemoglobin levels. [11] The relationship between erythropoietin and anemia in SCD has been previously documented in an American SCD cohort. [27]

Previous uncontrolled trials of iron supplementation in SCD have shown moderate responses in hemoglobin to iron supplementation in children with possible iron deficiency, defined as either low transferrin saturation, low MCV for age or low ferritin. [28,29] Limited case reports have suggested that relative iron deficiency in adults may be clinically beneficial by decreasing rates of sickling episodes due to lower per cell hemoglobin concentration. [16] α–thalassaemia is also hypothesized to be protective in this manner, by reducing concentration dependent polymerization of HbS. [30] Thus, lower iron status could counter-intuitively result in higher total hemoglobin in SCD. Therefore, it would be essential to carefully monitor the effects of giving iron to assess the relative advantages and risks. Targeting young children, who are most at risk of iron deficiency from diets low in bioavailable iron and low iron stores, at birth would seem sensible. Additionally, these children should also be receiving penicillin prophylaxis and effective anti-malarials (in malaria endemic areas) to reduce a potential effect of an increased risk of infection and its effects with and without hydroxyurea assessed. [3,31]

In conclusion, we have shown that in children with SCD, lower hepcidin levels are associated with lower steady- state hemoglobin, independent of EPO or reticulocyte % and markers of inflammation. Factors significantly associated with hepcidin levels were serum ferritin and CRP, although neither of these were independently associated with anemia. Future studies are required to further characterize the relationship between hepcidin, erythropoiesis and anemia, and to directly assess the effect of iron supplementation in SCD on hepcidin levels and iron absorption with a view to establish its role in the management of SCD and possible iron deficiency.

## Declaration of Interests

The authors declare no competing financial or other interests.
